# Relationship between Breastfeeding and Obesity in Childhood

**DOI:** 10.3329/jhpn.v30i3.12293

**Published:** 2012-09

**Authors:** Mohammadreza Vafa, Nazanin Moslehi, Shirin Afshari, Aghafatemeh Hossini, Mohammadreza Eshraghian

**Affiliations:** ^1^Nutrition Department, School of Public Health, Tehran University of Medical Sciences, Tehran, Iran; ^2^Endocrine Research Center, Firouzgar Hospital, Tehran University of Medical Sciences, Tehran, Iran; ^3^Biostatistics Department, School of Management, Tehran University of Medical Sciences, Tehran, Iran; ^4^Biostatistics Department, School of Public Health, Tehran University of Medical Sciences, Tehran, Iran

**Keywords:** Birthweight, Breastfeeding, Childhood, Infant-feeding, Introduction of complementary feeding, Obesity, Iran

## Abstract

A cross-sectional study was conducted to investigate the weight status and the relationship of infant-feeding variables, birthweight and birth order with BMI in a group of Iranian children. Five hundred and eleven students of both sexes at the first grade in elementary schools (aged 7 years) were recruited randomly from all 19 educational districts of Tehran. Weights and heights of children and their mothers were measured. Data on breastfeeding (BF), formula-feeding, the timing of introduction of complementary foods (CF), birthweight, and birth order were collected from the mothers. The 2007 WHO reference value was used for determining child's weight status. Regression analysis in single and a 2-level linear regression models was used for examining the independent relationships of infant-feeding variables, birthweight and birth order with childhood BMI. The prevalence of underweight and overweight in this group of children was 7.6% and 19.7%, respectively. Total time of BF and duration of exclusive BF were not associated with childhood BMI. The timing of introduction of CF was inversely related to childhood BMI after controlling for other variables (β:-0.34; 95% CI:-0.58,-0.10). Children with an early introduction of CF had significantly higher mean BMI (p for linear trend=0.012). Birth order and birthweight were related to childhood BMI significantly. These data suggest that overweight and obesity are nutritional problems among 7 years old Teharani children. The timing of introduction of CF, birth order, and birthweight were independent predictors of childhood BMI. Neither total time of BF nor duration of exclusive breastfeeding was associated with adiposity in children.

## INTRODUCTION

The prevalence and intensity of childhood obesity have been increasing with an alarming rate globally, and it is now considered to be at epidemic level. This nutritional problem has become an issue of international concern, which is common not only in developed but also in developing countries (1,2). It is associated with mortality and morbidity, including hypertension, cardiovascular, gastrointestinal, endocrine, respiratory and orthopaedic diseases as well as psychosocial problems both in adults and children. Obesity and its consequences account for huge costs for health and social care (3). While the treatment for obesity is time-consuming and inconclusive, the prevention of obesity is the most effective solution (3,4), which is impossible without understanding the risk factors of obesity. Knowing that childhood obesity is an important predictor of obesity in adulthood (5), special attention must be focused on prevention during childhood.

Recently, much of the research work has been conducted to investigate the possible effects of early nutritional experiences during foetal stage and infancy on later-life obesity. There is evidence that breastfeeding (BF) may have protective effect against obesity (6-9). In some studies, a time-dependent association between duration of BF and obesity has also been shown (10,11). However, more recent studies have reported null effects (12-14). Although the time when complementary foods (CF) are introduced has a potential effect on childhood obesity, its relationship with obesity is not still clear (15). The relationship of birthweight and later obesity has been examined in a large number of studies. In most studies, high birthweight was associated with obesity but in some cases not (16).

Most studies of childhood obesity focused on the relationship of potential risk factors with the prevalence of obesity, using BMI as a binary variable (obese vs non-obese). While the risk of co-morbidity may increase continuously across the whole spectrum of childhood BMI (17), it is also important to establish the association of these putative risk factors with BMI as continuous variable. Thus, we used BMI as a continuous variable in this study.

In Iran, meagre data on childhood obesity are available. There have also been very few studies that verified the association between foetal growth and infant-feeding with obesity. On the other hand, Iran has experienced a rapid nutritional transition and a decrease in BF rates, which can affect nutrition status and obesity (18,19). Therefore, we conducted a cross-sectional study to determine the nutrition status in a group of Iranian children aged 7 years (first-graders in elementary schools) based on their BMI, and then the association between infant-feeding and childhood BMI were investigated.

## MATERIALS AND METHODS

### Study design and sampling

This cross-sectional study has been conducted on children aged 7 years in Tehran, the capital city of Iran, in 2008. A random sample of 511 healthy children was selected in a multistage sampling technique from government elementary schools. Two schools from each of the 19 educational districts of Tehran (one for girls and the other for boys) were selected at random, except a district from where 4 schools were chosen. Thus, 40 schools were enrolled in this study. In the second stage, first grade students were selected randomly from each of the 40 schools. Twins, the multifoetal, and children with specific diseases were not included in this study.

Mothers were invited to the schools through a letter. The purposes and the methodology of research were explained to them by members of the study team, and written consent was obtained from them. All mothers participated in the study voluntarily, and the response rate was 100%. The study was approved by ethics committee of the School of Public Health in Tehran University of Medical Sciences.

### Anthropometric measurements

Weights and heights of the students and their mothers were measured by the authors and trained nutritionists. Body-weights were measured with light clothing and without shoes, using a portable analogue Seca Scale. Heights were measured in standing position, using a fixed tape meter to the nearest 0.5 cm. BMIs were also calculated using the formula kg/m^2^. The 2007 World Health Organization (WHO) growth reference was used for determining child's weight status. Maternal BMIs were categorized into 4 groups as underweight (<18.5), normal weight (18.5-24.9), overweight (25-29.9), and obese (≥30).

### Infant-feeding variables, birthweight, birth order, and parental characteristics

Infant-feeding variables, birthweight and birth order, and parental characteristics were obtained from the mothers, using a questionnaire. Mothers were first asked what type of feeding—BF, formula-feeding, or both (categorized as mixed feeding)— had been chosen. They were asked up to what age the child was exclusively breastfed and when the child completely stopped BF. In exclusive BF, the infant only received breastmilk without any addition of other liquid or solid feeds. Information on the ages (in month) when complementary food was introduced was also collected. We also asked the mothers whether their babies were on regular formula-feeding and, if so, what the total duration of formula-feeding was. Parental education was recorded as a socioeconomic status (SES) indicator.

### Statistical analysis

Child's weight status was determined according to the 2007 WHO reference and using descriptive statistics. The associations of infant-feeding variables, birthweight, birth order, and parental characteristics with child's BMI (as categorical variables) were tested by chi-square. In these analyses, the term ‘overweight’ included both overweight and obese children.

The associations of infant-feeding variables, birthweight, and birth order with child's BMI were also analyzed using univariate linear regression and multiple linear regression. Multiple linear regression included all variables with p<0.2 in univariate analysis plus gender, maternal BMI, and parental education. In linear regression models, all variables were entered as continuous variables, except parental education which was included as categorical variable with dummy coding. All continuous independent variables were checked for multicollinearity, and these were standardized. A test for linear trend was also conducted for CF after adjusting for other variables. Data were analyzed in SPSS (version 15) (SPSS, Chicago, IL, USA). A 2-level random effect linear regression model, taking students as level 1 and schools as level 2, was also executed with MLwin 2.02 to re-analyze the data for taking into account the dependency between BMI responses of children, which were nested in one particular school. The p value of <0.05 was considered to be the level of statistical significance.

## RESULTS

### Child's weight status

A total number of 511 students were recruited for this study from 40 schools; 54% were girls, and 46% were boys. The mean weight and height of students were 23.83±5.04 kg and 121.88±5.67 cm respectively; 7.6% of the children were underweight, and the prevalence of overweight and obesity was 8% and 11.7% respectively. There was no association between child's weight status and gender [χ^2^ (4, n=511)=4.26, p=0.37]. The prevalence of overweight and obesity was higher in girls than boys (22.1 vs 17.1). However, it is not statistically significant [χ^2^ (1, n=511)=1.64, p=0.20].

### Infant-feeding variables, birthweight, birth order, and child's BMI

Approximately 96.1% of the children had been ever breastfed; 20.3% of the children were breastfed for less than 12 months (Table 1).

The associations between BMI (as categorical variable), infant-feeding, birghtweight, and birth order are presented in Table 2. Type of nutrition [χ^2^ (2, n=511)=3.78, p=0.151], total duration of BF [χ^2^ (6, n=508)=8.11, p=0.23], duration of exclusive BF [χ^2^ (4, n=503)=6.22, p=0.184], total time of formula-feeding [χ^2^ (2, n=510)=2.31, p=0.315], and timing of introduction of CF [χ^2^ (4, n=503)=6.22, p=0.186] were not significantly associated with child's weight status. The prevalence of overweight by birthweight was significantly different [χ^2^ (4, n=510)=15.99, p=0.003]. The prevalence of overweight was higher in children with birthweight of more than 3500 g than the other 2 groups. The prevalence of overweight was also higher in children with birthweight of 2801-3500 g than children with birthweight of ≤2800 g. Birth order was not significantly related to child's weight status [χ^2^ (4, n=504)=4.74, p=0.315].

**Table. 1.** Infant-feeding, birth and parental characteristics of participants

Characteristics	Frequency	Percentage
Type of nutrition	511	
Breastmilk	387	75.7
Formula milk	20	3.9
Both	104	20.4
Total time of BF (months)	508	
≤6	60	11.8
>6-12	43	8.5
>12-18	67	13.2
>18	338	66.5
Duration of exclu-sive BF (months)	503	
≤1	95	18.9
>1-4	92	18.3
>4	316	62.8
Total time of formu-la-feeding (months)		510
≤6	404	79.2
≤6	106	20.8
Timing of introduction of CF (months)		503
≤4	51	10.1
>4-6	333	66
>6	119	23.9
Birthweight (g)	510	
≤2,800	81	15.9
2,801-3,500	334	65.5
>3,500	95	18.6
Birth order	504	
First	244	48.4
Second	158	31.3
Third and higher	102	20.2
Maternal weight BMI category (BMI, kg/m2)	506	
Underweight (<18.5)	4	0.8
Normal (18.5-24.9)	161	31.8
Overweight (25-29.9)	209	41.2
Obese (≤30)	133	26.1
Maternal education (years)	510	
≤5	51	10
5-12	362	71
>12	97	19
Paternal education(years)	510	
<5	52	10.2
5-12	307	60.2
>12	151	29.6

**Table. 2.** Infant-feeding, birthweight, birth order by BMI category

Variable	BMI category			p value
	Underweight n (%)	Normal n (%)	Overweight n (%)	
Type of nutrition				
Breastmilk	30 (7.8)	288 (74.4)	69 (17.8)	0.151
Formula milk or both	9 (7.3)	83 (66.9)	32 (25.8)		
Total time of BF (months)				
≤6	4 (6.7)	38 (63.3)	18 (30)	0.230
>6-12	5 (11.6)	29 (67.4)	9 (2.9)	
>12-18	7 (10.4)	51 (76.1)	9 (13.4)	
>18	22 (6.5)	252 (74.6)	64 (18.9)	
Duration of exclusive BF (months)				
≤1	59 (5.3)	69 (72.6)	21 (22.1)	0.184
>1-4	3 (3.3)	67 (72.8)	22 (23.9)	
>4	30 (9.5)	230 (72.8)	56 (17.7)	
Total time of formula-feeding (months)				
≤6	29 (7.2)	300 (74.3)	75 (18.5)	0.315
>6	9 (8.5)	71 (67)	26 (24.5)	
Timing of introduction of CF (months)				
≤4	1 (2)	36 (70.6)	14 (27.5)	0.186
>4-6	24 (7.2)	242 (72.7)	67 (20.1)	
>6	12 (10.1)	89 (74.8)	18 (15.1)	
Birthweight (g)				
≤2,800	14 (17.3)	57 (70.4)	10 (12.3)	0.003
2,801-3,500	21 (6.3)	246 (73.7)	67 (20.1)		
>3,500	4 (4.2)	67 (70.5)	24 (25.3)		
Birth order				
First	17 (7)	172 (70.5)	55 (22.5)	0.315
Second	12 (7.6)	114 (72.2)	32 (20.3)	
Third and higher	10 (9.8)	79 (77.5)	13 (12.7)	

In univariate model, variables related to child's BMI with p<0.2 were: duration of exclusive breastfeeding, total time of formula-feeding, timing of introduction of CF, birthweight, and birth order. Child's BMI increased with total time of formula-feeding (p=0.040) and birthweight (p=0.001). It decreased with timing of introduction of CF (p=0.007) and tended to decrease with duration of exclusive BF (p=0.112) and birth order (p=0.161). In multiple linear regression, after controlling for gender, maternal BMI, and parental education, timing of introduction of CF was the only infant-feeding variable significantly associated with child's BMI (p=0.006). Birthweight and birth order were also significantly related to child's BMI (p=0.004 and p=0.013, respectively) (Table 3). When we entered CF as a categorical variable, inverse association between CF and child's BMI was found (p for linear trend=0.012). The mean BMI in children with the time of CF >4-6 (β:-0.76; 95% CI: −1.52,-0.002) and >6 months (β:-1.15; 95% CI: −2.04,-0.27) were lower than in children with early introduction of CF (≤4). The multilevel analysis revealed that variances of the first- and second-level residuals were 5.84±0.397 and 0.199±0.169 respectively, indicating intraclass correlation (ICC) equal to 0.033. Wald test showed that variance of second-level residual did not have significant variation, after adjusting for independent variables (p=0.08). Also, two-level random effect model did not have significant improvement in −2 log likelihood compared to single-level model (change in −2 log likelihood=1.92, df=1, p=0.17), So, all results are based on single-level multiple linear regression model.

**Table. 3.** Associations of infant-feeding variables, birthweight, and birth order with child's BMI

Independent variable	Univariate linear regression			Multiple linear regression¶		
β	95% CI	p value	β	95% CI	p value
Total time of BF (months)‡	-0.07	-0.29,0.16	0.548			
Duration of exclusive BF	-0.18	-0.41,0.04	0.112	0.08	-0.19,0.34	0.576
Total time of formula-feeding (months)	0.23	0.01,0.46	0.040	0.20	-0.05,0.46	0.117
Timing of introduction of CF	-0.31	-0.54,-0.09	0.007	-0.34	-0.58,-0.10	0.006
Birthweight (per 100 g)	0.38	0.16,0.60	0.001	0.32	0.10,0.54	0.004
Birth order	0.16	-0.39,0.06	0.161	-0.28	-0.50,-0.06	0.013

¶Multiple regression includes all variables with p<0.2 in univariate model plus gender, maternal BMI, and parental education.

‡All continuous independent variables are in standardized form

## DISCUSSION

In this study, the prevalence of underweight was 7.6%, and the combined prevalence of overweight and obesity was 19.7% in Tehrani children aged 7 years. The child's weight status was similar in both sexes. Despite the higher rates of overweight and obesity in girls, there were no significant differences between the sexes (22.1 vs 17.1). These findings show that childhood obesity is a nutritional problem in Tehrani children. The prevalence of childhood obesity varies across populations (20). Cut-off values and type of reference to define obesity, the age of participants, and the time when the sample was examined differ in studies reporting prevalence of obesity, making it difficult to compare the rate of childhood obesity among populations. In the present study, the 2007 WHO growth reference was used for the calculation of prevalence because it was the most relevant BMI reference to define obesity (21). This reference is also recommended for international use and has a potential to be the future international reference for the surveillance of overweight and obesity (22).

We also investigated the associations of infant-feeding practices and infant variables with childhood BMI at 7 years of age as early-life predictors of adiposity. In the present study, timing of introduction of CF was significantly and inversely associated with childhood BMI. There was also a significant linear trend for the time of CF; children having CF at >4-6 months and >6 months had significantly lower BMI than children having early CF (≤4 months) (p for trend=0.012), after controlling for other variables. The time of introduction of CF is a period of particular vulnerability; however, the association between this time and later risk of obesity is not still clear. Few researches reported the time of introduction of CF and adiposity at 7 years. Wilson *et al.*, in their Dundee cohort of 674 infants, showed that, in infants with early introduction of CF (<15 weeks), weight and percentage of body fat increased significantly at 7 years of age than the ones with late introduction of CF (≥15 weeks) (23). Reilly *et al.*, in the Avon longitudinal study of parents and children, reported that the age of introduction of CF was not a significant risk factor of obesity at 7 years of age (13). Other studies that explored the effect of age at introduction of CF on later adiposity in different ages also provided inconsistent results (6,12,24). In a recent systematic review of association between timing of introducing solid food and obesity in infancy and childhood, no clear association was found. In that systematic review, only studies of participants living in developed countries were included; so, it was not applicable to other populations (15).

In our study, the lower prevalence of overweight in breastfed children than formula- or mixed-fed children was not statistically significant. We also did not find a protective effect of BF, neither as total time of BF nor duration of exclusive BF. These findings are consistent with those from some previous observational studies (12-14) but not from others (6-9). Randomized controlled trial of breastfeeding (PROBIT) also showed that promotion of BF increased the duration and exclusivity of BF but did not reduce BMI and other measures of adiposity at the age of 6.5 years (25). Meta-analyses concluded an inverse association between BF and risk of obesity at different ages; however, adjustment for confounders attenuated or abolished these associations (11,26,27). In a meta-analysis of the effect of breastfeeding on mean BMI throughout life, mean BMI was found slightly lower among breastfed subjects. Adjustment for socioeconomic status, maternal BMI, and maternal smoking eliminated the effect (28). Thus, the protective effect of BF reported in some studies may be due to inadequate control of confounding factors or publication bias (25,28,29). On the other hand, the protective effects of breastfeeding on childhood obesity are mostly reported in developed western populations whereas non-European researchers showed the null effect. This discrepancy pattern in findings suggests that the apparent protective effects of BF may be due to socially-patterned confounders (14).

It has been suggested that the null effects of BF on childhood BMI, using BMI means or linear regression models, may be due to different effects of breastfeeding on different BMI distributions. This situation could happen when prevalence of overweight and underweight decreased concomitantly, and so the mean BMI remained unchanged (30). However, when we treated BMI as binary variable (obese vs non-obese), logistic regression also showed no significant association between breastfeeding and obesity.

There is also evidence that the relation of BF with BMI differed with age. In some studies, the inverse relationship between breastfeeding and BMI at 1 year of age attenuated at the age of 7 years or even disappeared later in childhood or adulthood (24,31,32). Therefore, it is possible that other genetic and environmental factors, such as dietary patterns, sociocultural and economic status, and parental characteristics, diminish or undo the effects of breastfeeding on childhood BMI after 1 year of age.

In our study, birth order was inversely associated with childhood BMI, after controlling for infant-feeding variables, birthweight, and parental characteristics. There was a significant increase in child's BMI with increase in birthweight, adjusting for infant-feeding and birth order. This finding is in agreement with most researches that reported association of birthweight with obesity (16). In the Avon longitudinal study of parents and children, birthweight was also reported as one of the independent risk factors for childhood obesity at the age of 7 years (13). In a prospective birth cohort study in China, birthweight was also linearly and positively associated with childhood BMI at 3-6 years of age, independent of maternal BMI and other confounders (33). Thus, it seems that the relationship between birthweight and child's BMI is independent of maternal BMI and the socioeconomic status of the samples. Genetic factors and early programming by the environment *in utero* may explain the association between high birthweight and the risk of later obesity (33,34).

The high rate of breastfeeding with less social patterning is one of the strengths of our study. Thus, the results may be less confounded by social patterning of breastfeeding. In this study, duration of exclusive breastfeeding and total time of breastfeeding were investigated apart from the comparison of breastfed with non-breastfed or mixed-fed children. We also determined different infant-feeding variables, and the independent association of each feeding variable was illustrated, after controlling for other feeding variables, birthweight, birth order, and parental characteristics.

### Limitations

Our study has several limitations. First, the cross-sectional design precludes inferences about the role of breastfeeding. Second, this design of the study is prone to be confounded by many environmental factors; however, we cannot rule out residual confounding by other unmeasured or unknown confounders. Third, data on infant-feeding variables, birthweight, and birth order were collected based on mother's recall, which are suspected to have recall bias. Collecting data many years later can lead to misclassified exposures and consequently inappropriate conclusions. Fourth, we used only BMI as the measure of adiposity but it does not distinguish between fat and lean mass. We did not find any differences in BMI by BF status; however, its effects on body composition are not evident. Fifth, body-weight at the time of introduction of CF may be a potential confounder because heavier infants are introduced to CF earlier (35,36). However, in the analyses of association between CF and childhood BMI, we could not take into account the body-weight at the time of CF.

### Conclusions

We observed high rates of overweight and obesity in Tehrani children at 7 years of age. Preventive strategies are needed to prevent further increases in the prevalence of overweight and obesity. The time of introduction of CF, birthweight, and birth order were independent predictors of childhood BMI. Breastfeeding (either total time of BF or duration of exclusive BF) was not associated with child's BMI at this age-group. Further studies are needed to explore the independent determinants of BMI in Iranian children to develop effective and culture-sensitive prevention strategies.

## ACKNOWLEDGEMENTS

We thank the participants in the study for their enthusiastic support. This study has been supported by a grant from the Tehran University of Medical Sciences, and we appreciate the Vice Chancellor of Research of the University for supporting this study.
